# Deficiency of glycogen synthase promotes lipid accumulation through ChREBP and AKT-mTOR1-SREBP1 axis activation in mice

**DOI:** 10.1016/j.jlr.2025.100962

**Published:** 2025-12-15

**Authors:** Liangkui Li, Jianan Lang, Longyan Yang, Dong Zhao

**Affiliations:** Beijing Key Laboratory of Diabetes Prevention and Research, Center for Endocrine Metabolism and Immune Diseases, Beijing Luhe Hospital Capital Medical University, Beijing, China

**Keywords:** lipid, glycogen, glycogen synthase, glucose, ChREBP, SREBP1

## Abstract

Metabolic dysfunction-associated steatotic liver disease (MASLD) has become highly prevalent worldwide, largely as a consequence of the global obesity epidemic. This research endeavors to elucidate the role and molecular mechanisms of hepatic glycogen synthase (GS) in MASLD progression. Published transcriptomic data reveal a downward trend in GYS2 gene expression in patients with obesity, MASLD, and metabolic dysfunction-associated steatohepatitis. In mouse models of MASLD, GYS2 gene or protein expression was downregulated, consistent with the human data. Here, GS-deficient mice fed with a normal diet displayed hepatic lipid accumulation and liver injury, whereas hepatic steatosis progression and inflammation were aggravated in mice fed with a high-fat diet. Loss of hepatic GS stimulated fatty acid de novo synthesis through carbohydrate-response element-binding protein and AKT-mTOR1-sterol regulatory element-binding protein 1 axis pathways. In GS-deficient mice, lipid accumulation in the hepatocytes significantly decreased when carbohydrate-response element-binding protein and sterol regulatory element-binding protein 1 levels were suppressed to levels comparable to those of cytotoxic T lymphocyte hepatocytes. Forced expression of hepatic GS by adeno-associated virus in *db/db* mice ameliorated lipid accumulation in male mice. Our findings provide proof of concept whereby targeting glycogen metabolism in hepatocytes may offer potential therapeutic avenues to treat MASLD.

Metabolic dysfunction-associated steatotic liver disease (MASLD), formerly known as nonalcoholic fatty liver disease, is the most common chronic liver disease worldwide, affecting more than 30% of the world’s population (1, 2). It is closely associated with metabolic disorders, including obesity, dyslipidemia, insulin resistance, and type 2 diabetes (3). This close association underscores the complex metabolic nature of the disease and highlights the urgent need for novel therapeutic targets to address its multifaceted pathology (4, 5, 6).

In the postprandial state, elevated glucose and insulin levels activate multiple metabolic pathways, such as glycogenesis and lipogenesis. Specifically, the insulin-induced AKT-GSK3β signaling axis is considered a key pathway regulating glycogen synthesis and mediating glucose clearance (7, 8, 9, 10). Similarly, it promotes lipogenesis through signaling cascades that activate lipogenic transcription factors, including sterol regulatory element-binding protein 1 (SREBP1) and carbohydrate-response element-binding protein (ChREBP) (11).

Glycogen is the largest soluble cytosolic macromolecule and serves as the principal storage form of glucose (12, 13). Upon feeding, glucose is cleared from the bloodstream primarily by conversion to glycogen through a process called glycogenesis. Synthesis of glycogen is mainly accomplished by glycogen synthase (GS). There are two GS isoforms encoded by separate genes in mammals. *GYS1* is ubiquitously expressed in muscle and many other tissues, whereas *GYS2* is specifically expressed in the liver. The genetic disorder known as glycogen storage disease 0 is caused by the loss-of-function mutations in the *GYS2* gene (14). These patients display postprandial hyperglycemia, hyperlactatemia, and hyperlipidemia, indicating that liver glycogen synthesis during normal glucose disposal has a role in this disease. Similarly, mice lacking liver GS protein expression develop insulin resistance, hypoglycemia, and hepatic steatosis, suggesting the important role of GS in regulating both glucose and lipid homeostasis (15, 16). However, the underlying mechanism of liver GS in the development of MASLD remains unclear.

In this study, we explored the role of hepatic GS in a mouse model. We determined the molecular mechanisms underlying disordered lipid metabolism and inflammation induced by GS deficiency in the liver. Our work demonstrates that loss of liver GS activates the ChREBP and SREBP1 pathways. Taken together, these findings suggest a potential therapeutic strategy for MASLD.

## Materials and Methods

### Mouse models

Mice were housed in a temperature-controlled environment (22◦C, 12-h light-dark cycle) with free access to water and standard rodent chow diet, except as noted. All mice used for experiments were bred on the C57BL/6J background. Cre-dependent spCas9 knock-in mice were from Jackson Laboratory (17). All GS KO mice in this article were CRISPR-mediated KO in the spCas9 mice. The high-fat diet (HFD) was purchased from Research Diet (D12492, 60 kcal% fat). The animal study in this article was approved by the “Animal Experiments and Experimental Animal Welfare Committee of Capital Medical University.”

### Adeno-associated virus production and delivery

Adeno-associated virus (AAV) was packaged in human embryonic kidney 293T cells. In brief, AAV transfer plasmids, Rep/Cap(2/8) plasmids, and helper plasmids were transfected into cells using polyethyleneimine according to the manufacturer’s protocol. Cells were harvested at 48 h post-transfection. Virus purification and titer quantification were performed as described before (17). AAV serotype 8 (AAV8)-Cre-LacZ single guide RNA (sgRNA) or AAV8-Cre-Gys2 sgRNA was delivered into 6-week-old mice by tail vein injection. sgRNA sequences are listed in supplemental Table S1. 4E^11^ viral genome copies of pX602-AAV-Cre-sgRNA were used for the CRISPR-mediated acute KO gene in each mouse.

For overexpression experiments, AAV8 was used to express GS (human). AAV-thyroxine-binding globulin (TBG)-FLAG-hGYS2 was generated from AAV-TBG-GFP (Addgene; 105535) by replacing the GFP sequence with the respective complementary DNA (cDNA).

### Serum biochemical analysis

The levels of serum triglyceride (TAG) and NEFAs were determined using a serum TAG determination kit (Sigma) and Lab Assay NEFA kit (Wako Pure Chemical Industries, Japan) following the manufacturer's instructions, respectively. The serum insulin concentration was determined using Ultrasensitive Mouse Insulin ELISA Kit (90080, Crystal Chem). Serum cholesterol concentration was determined using CHO KIT (Biosino Bio-Technology and Science, Inc, 100000180). The serum levels of alanine transaminase (ALT) and aspartate transaminase (AST) were determined according to the manufacturer’s instructions (C009-2-1 for ALT, C010-2-1 for AST; NJJCBIO). Serum TNFα level (430907, BioLegend) and IL6 (431307, BioLegend) were quantified by an ELISA kit. Urinary albumin and creatinine were determined by Albumin Elisa Kit (ab108792, Abcam) and Creatinine Assay Kit (MAK080-1 kit, Sigma), respectively. Serum blood urea nitrogen (BUN) was determined separately using commercial assay kits from Nanjing Jiancheng Bioengineering Institute (Nanjing, China) according to the manufacturer’s instructions.

### Fat/lean mass and metabolic cage

Fat mass and lean mass were measured using EchoMRI 100&A10 (Echo MRI). Briefly, using the PhenoMaster/LabMaster System (TSE Systems GmbH, Bad Homburg, Germany), four mice were randomly chosen from each group and individually placed into metabolic cages to measure O_2_ consumption, respiratory exchange rate, food intake, and drink intake. In addition, 4-month-old mice were individually monitored for 48 h with data collection at intervals of 27 min after 1 day of adaptation.

### Glucose tolerance test and insulin tolerance test

The mice were fasted for 12 h prior to intraperitoneal injection with glucose (1 g glucose in PBS/kg mouse) for the glucose tolerance test (GTT) assay. Separately, mice were intraperitoneally injected with insulin (1 U insulin in PBS/kg mouse) after 6 h fasting for the insulin tolerance test (ITT) assay. Blood glucose levels were determined with a blood glucose monitoring system (ACCU-CHEK Advantage II; Roche, Switzerland) using blood collected from the tail vein.

### RT-PCR

Total RNA was extracted from mouse liver tissues using TRIzol (Thermo Fisher) according to the manufacturer's instructions. First-strand cDNA synthesis was conducted with Revert Aid First Strand cDNA Synthesis Kit (Thermo Scientific). RT-PCR was performed to detect gene expression levels using Power SYBR Green PCR Master Mix (Applied Biosystems) on an ABI 7500 (Applied Biosystems) with a reaction volume of 20 μl. Beta-actin gene was used as a reference gene. The primer sequences used are listed in supplemental Table S2.

### Measurement of liver lipid content

Isolation and measurement of total lipid from mouse tissues have been previously described with minor modifications (18, 19). Briefly, tissues (nearly 30 mg liver) were homogenized in PBS buffer. A chloroform/methanol (v/v 2:1) solution was rapidly added to the homogenate, and the samples were vortexed thoroughly. After centrifugation at 1,000 *g* for 5 min, the upper organic phase was collected and dried under nitrogen gas. Lipids were resuspended in chloroform containing 1% Triton X-100 (1 ml) and subsequently dried under a stream of nitrogen gas. Finally, lipids were resuspended in water (200 μl). Isolated lipids were diluted for detection accordingly. TAG and FFA levels were measured using TAG reagent (Sigma-Aldrich) and free glycerol reagent (Sigma-Aldrich) and a Lab Assay NEFA kit (Wako Pure Chemical Industries, Japan), respectively.

### Determination of glucose and glycogen levels

The glucose and glycogen levels of mouse tissues were measured as previously described (20). Briefly, 30 mg of liver tissues or 50–100 mg of other tissues were homogenized in 1 ml precooled 6% perchloric acid (Sigma-Aldrich), followed by centrifugation at 14,000 *g* at 4°C for 10 min. The supernatant (S1) was neutralized with KOH and centrifuged. The resulting supernatant (S2) was collected. Next, 20 μl of S2 was added to 100 μl of 1 mg/ml amyloglucosidase (Sigma-Aldrich, A7420) in 0.2 M acetate buffer (pH 4.8) for digestion at 50°C for 2 h. After digestion, the solution (S3) was taken for glucose measurement by a glucose assay kit (Sigma-Aldrich, GAGO20). The glycogen level was calculated by subtracting the basal glucose level in S2 from that of S3.

### Metabolomics analysis of the liver

Extraction of metabolites from liver tissues has been previously described (21). Briefly, 500 μl of 80% (v/v) HPLC-grade methanol (cooled to −80°C) was added to frozen tissue pieces (nearly 40 mg liver) and ground for 1–2 min on dry ice. After centrifugation at 13,000 *g* for 10 min, the supernatant of the tissue homogenates was transferred to a new Eppendorf tube and stored at −80°C. Four hundred microliter of 80% (v/v) methanol (−80°C) was added to the remaining precipitate and vortexed for 1 min at 4–8°C. After centrifugation at 13,000 *g* for 10 min, the supernatant was transferred and combined from both extractions. The combined supernatants were dried to a pellet using no heat. The metabolites were then further analyzed by LC-MS/MS (22).

### Protein expression by immunoblotting analyses

The frozen tissues were homogenized in a lysis buffer (20 mM Tris-HCl, 150 mM NaCl, 1 mM EDTA, 1mM EGTA, 1% Triton X-100, and protease inhibitor, pH 7.4) and centrifuged for 20 min at 10,000 *g* to discard cell debris. The total protein concentrations were determined using a Bio-Rad protein assay kit. The proteins were subjected to Western blot analysis with the indicated antibodies. Antibodies against GS (22371-1-AP) and p70 S6 kinase (14485-1-AP) were purchased from Proteintech. Antibody against ChREBP (NB400-135, 1/1000e) was purchased from Novus. Antibodies against Gpat3 and anti-Gpat4 were purchased from PTG (Wuhan, China). Antibodies against AKT (9272), Phospho-AKT (Ser473) (9271), GSK-3β (12,456), phospho-GSK-3β (Ser9) (9336), SCD-1 (2438), ACC1 (3676S), and phospho-p70 S6 kinase (97596) were purchased from Cell Signaling Technology. Antibodies against SREBP1 (sc-13551), DGAT1 (sc-26173), and DGAT2 (sc-32400) were purchased from Santa Cruz Biotechnology. Antibodies against Fasn (ab128870) were purchased from Abcam. Antibodies against TUBULIN (T0198) and FLAG (F1804) were obtained from Sigma-Aldrich.

### Isolation of primary hepatocytes

Primary hepatocytes were isolated from the livers of 10-week-old male mice, 2 weeks after AAV injection. Mice were anesthetized with 2.5% avertin and dissected. Through the portal vein, the liver was perfused with 50 ml Krebs Ringer with glucose buffer to remove residual blood and subsequently perfused with 30 ml Krebs Ringer with glucose buffer containing 25 mg type IV collagenase (C5138, Sigma) until the liver became soft. The liver was immediately removed, cut into pieces, and filtered through a 70-mm membrane (Millipore) to remove tissue debris. The hepatocytes in the filtered supernatant were collected and washed three times with cold DMEM by centrifugation at 50 *g* for 5 min. The isolated hepatocytes were suspended in DMEM containing 10% FBS and 1% penicillin-streptomycin.

### Cell culture

Primary hepatocytes were cultured in DMEM (Invitrogen) containing 10% FBS (Invitrogen), 2 mM l-glutamine, 100 U/ml penicillin, and 100 mg/ml streptomycin at 37°C in a humidified incubator containing 5% CO_2_. All cells utilized were tested negative for mycoplasma. Subsequently, primary hepatocytes were starved overnight, followed by insulin and glucose stimulation.

### RNA interference

siRNAs were introduced into primary hepatocyte by Lipofectamine™ 3000 (L3000001; Invitrogen™). Cells were harvested after 48 h. The knockdown efficiency for each protein was evaluated by Western blot analysis. siRNAs used in all experiments are listed in supplemental Table S3.

### Histological analysis

Livers from mice were excised and fixed in 10% formalin buffer, dehydrated, embedded in paraffin blocks and sectioned with a thickness of 5 μm. The sections were stained with H&E. Livers from mice were excised, fixed in 10% formalin buffer, and sectioned. Oil Red O staining, Periodic acid-Schiff (PAS), or immunohistochemistry assay were performed according to the standard protocols.

### Statistics

All statistical analyses were performed in GraphPad Prism, version 5 (https://www.graphpad.com, GraphPad Software). Data are presented as mean values ± SEM. Data were analyzed using a two-tailed Student’s *t*-test. ∗*P* < 0.05, ∗∗*P* < 0.01, and ∗∗∗*P* < 0.001.

## Results

### GYS2 or GS is downregulated in patients with metabolic dysfunction-associated steatohepatitis and MASLD-mouse model

**Fig. 1:**
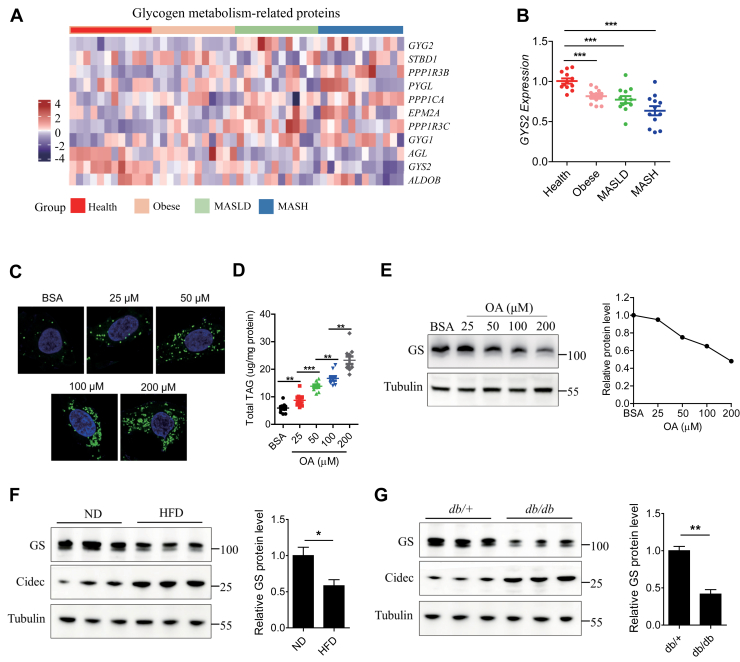
GYS2 or GS is downregulated in patients with MASH and MASLD mouse model. A: Hepatic gene expression analysis of 12 healthy participants, 12 patients with obesity, 12 patients with MASLD, and 12 patients with MASH, from the GSE126848 dataset. B: Relative Gys2 mRNA levels of healthy (n = 12), obesity (n = 12), MASLD (n = 12), and MASH (n = 12) in GSE126848. C: Representative Bodipy-LD images of HepG2 cells treated with indicated concentrations of OA. D: Cellular TAG content of panel C. E: Representative Western blots (left panel) and quantitative analysis (right panel) of GS proteins harvested from HepG2 cells with indicated treatments. F: Representative Western blots (left panel) and quantitative analysis (right panel) of GS expression in the livers of 22-week-old male C57BL/6J mice fed with 16 weeks of ND or HFD, starting from when mice were 6 weeks old (n = 3/group). G: Representative Western blots (left panel) and quantitative analysis (right panel) of GS expression in the livers of 16-week-old male *db*/*+* or *db*/*db* mice (n = 3/group). Data are presented as mean ± SEM. Data were analyzed using a two-tailed Student’s *t*-test. ∗*P* < 0.05, ∗∗*P* < 0.01, and ∗∗∗*P* < 0.001.

To identify potential glycogen metabolism-related proteins implicated in the pathogenesis of MASLD, we first examined hepatic gene expression in the published transcriptome dataset (Gene Expression Omnibus: GSE126848) from 12 healthy participants, 12 patients with obesity, 12 patients with MASLD, and 12 patients with metabolic dysfunction-associated steatohepatitis (MASH) (23) (Fig. 1A). Notably, GYS2 gene expression displayed a gradual reduction in patients with obesity, MASLD, and MASH (Fig. 1B). The strongest reduction was observed in patients with MASH (∼40%).

Next, in order to mimic liver steatosis in patients with MASLD, oleic acid (OA) was added to HepG2 cells cultured in vitro (24). As expected, increasing OA concentration induced an increase in the number of lipid droplets (LDs) in HepG2 cells, accompanied by a corresponding elevation in TAG content (Fig. 1C and D). Interestingly, GS protein levels were downregulated in a dose-dependent manner during 24 h OA stimulation (Fig. 1E). The changes in *GYS2* mRNA and GS protein expression were also observed in mice fed with 16 weeks of HFD (Fig. 1F and supplemental Fig. S1A). Here, *GYS2* mRNA and GS protein were significantly decreased relative to the normal diet (ND)-fed mice (Fig. 1F and supplemental Fig. S1A). Similarly, *GYS2* mRNA and GS protein levels also were also downregulated in the liver of *db/db* mice compared with *db/+* mice (Fig. 1G and supplemental Fig. S1B). These data highlight a potential role of hepatocyte-derived GS in the progression of MASLD.

### Hepatic GS deficiency promotes lipid accumulation and inflammation

**Fig. 2:**
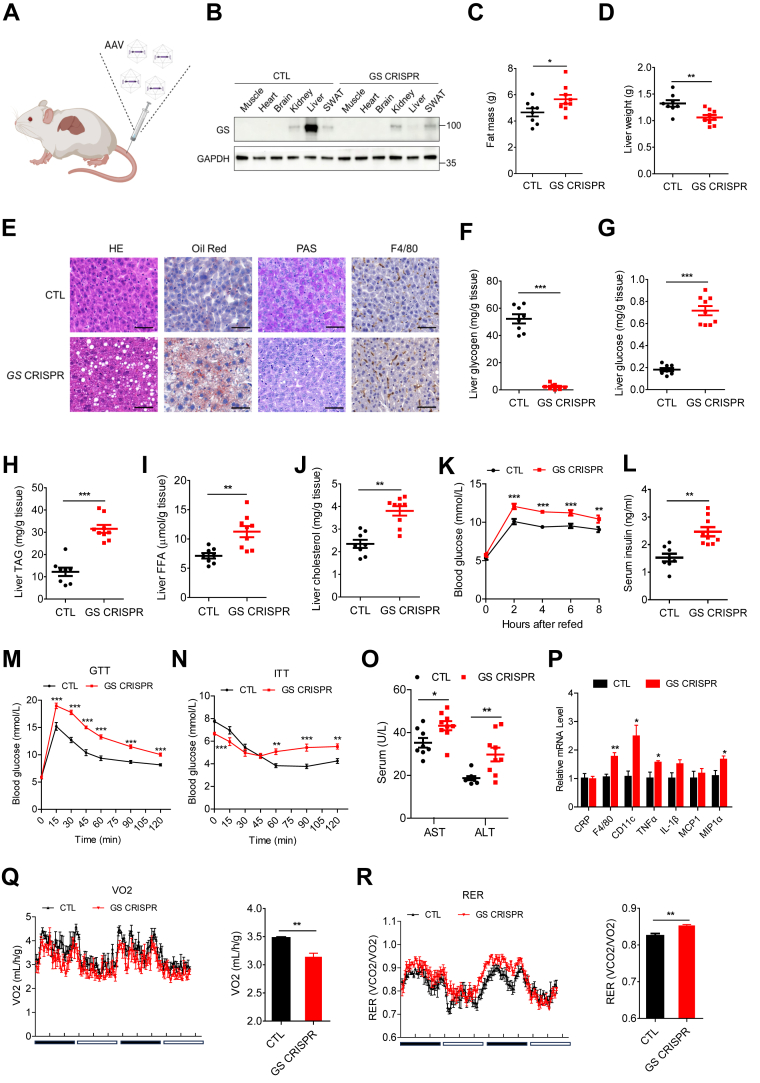
ND-fed GS-deficient mice exhibit a distinct hepatic lipid accumulation phenotype. A: Schematic drawing of CRISPR-mediated acute hepatic gene inactivation on ‘‘silent’’ spCas9 knock-in mice. B: Representative Western blots of tissues from mice receiving either control gRNA or GS targeting gRNA with the indicated antibodies. Hepatic GS is efficiently depleted in GS CRISPR mice. C: Fat mass of CTL and GS-deficient mice (n = 8). D: Liver weight of CTL and GS-deficient mice (n = 8). E: Representative images of liver sections stained with H&E, Oil Red O, PAS, or F4/80. The scale bars represent 50 μm. F–J: Determination of glycogen, glucose, total TAGs, FFAs, and total cholesterol in liver tissues (n = 8). K: Overnight-fasted CTL and GS-deficient mice were refed, and blood glucose was measured at the indicated time points (n = 8). L: Serum insulin measurement of overnight-fasted CTL and GS-deficient mice at 8 h postprandial (n = 8). M: GTT (n = 8); N: ITT (n = 8); O: serum AST and ALT (n = 8) measurements. P: Quantitative RT-PCR analysis of liver mRNA expression (n = 6). Q: Oxygen consumption (VO2). R: Respiratory exchange rate (RER) of CTL and GS-deficient mice monitored for 48 h. Dark phase refers to 8 PM to 8 AM (next day), whereas light phase refers to 8 AM to 8 PM (n = 4 mice per group). Data are presented as mean ± SEM. Data were analyzed using a two-tailed Student’s *t*-test. ∗*P* < 0.05, ∗∗*P* < 0.01, and ∗∗∗*P* < 0.001.

To investigate the function of GS in vivo, we performed AAV-mediated in vivo gene editing to specifically inactivate GS in the liver (Fig. 2A) using a mouse line bearing a ‘‘silent’’ spCas9 transgene (17). In these mice, hepatic GS was effectively knockdown as shown with GS immunoblotting and RT-PCR analysis of *GYS2* mRNA in the liver lysates (Fig. 2B and supplemental Fig. S2A). Under ND, the body weight and lean mass remained similar, whereas the fat mass was slightly but significantly increased in the hepatic GS-deficient mice, compared with the controls (Fig. 2C and supplemental Fig. S2B, C). Interestingly, the liver weight of hepatic GS-deficient mice was significantly lower, whereas the weight of the subcutaneous white adipose tissue (WAT) displayed an opposite trend (Fig. 2D and Sup Fig. 2D). Other tissues, including gonadal WAT, heart and kidney, showed no significant differences compared with control mice (supplemental Fig. S2D). Interestingly, a complete absence of glycogen deposits was observed upon GS inactivation by PAS staining (Fig. 2E). More precisely, hepatic GS-deficient mice exhibited a 94% depletion of liver glycogen (Fig. 2F). Glycogen content in muscle, heart, brain, kidney, and subcutaneous WAT did not display any substantial differences between control and GS-deficient mice (supplemental Fig. S2E). The lack of glycogen storage capacity was accompanied by ∼3 times increase in hepatic glucose levels in GS-deficient mice (Fig. 2G). Conversely, GS-deficient mice displayed an increase in fat accumulation in the liver as evidenced by the emergence of enlarged LDs visualized with HE and Oil Red O staining (Fig. 2E). Consistently, hepatic TAG, FFA, and cholesterol levels were also significantly elevated in GS-deficient mice by 157%, 58%, and 62%, respectively (Fig. 2H–J). In addition, GS-deficient mice also displayed higher serum TAG and NEFA compared with control mice in the fasting state (supplemental Fig. S2F). No significant difference was observed for serum cholesterol between both groups of mice in the fasting state (supplemental Fig. S2F). F4/80 histochemistry staining showed the presence of enhanced inflammation in GS-deficient mice compared with CTL mice (Fig. 2E).

To study the role of glycogen in blood glucose regulation, mice were fed with ND for 8 h after overnight fasting. Importantly, GS-deficient mice maintained higher blood glucose and insulin levels persistently after refeeding (Fig. 2K, L). Concomitantly, GTT and ITT showed a similar trend (Fig. 2M, N). Further blood chemistry analysis revealed an increase in serum AST and ALT levels in GS-deficient mice, indicating liver injury (Fig. 2O). Additionally, the mRNA levels of genes related to inflammation also increased slightly but significantly in these mice compared with control animals (Fig. 2P). The studies from the metabolic cage revealed that GS-deficient mice displayed lower oxygen consumption and higher respiratory exchange rate when compared with the controls, suggesting elevated glucose utilization, consistent with higher glucose levels in the liver and circulation despite no differences in food and water intake (Fig. 2Q, R and supplemental Fig. S2G, H). These data suggest that GS-deficient mice exhibit distinct hepatic fat accumulation, even when fed an ND. Of note, a comparable phenotype was also observed in female GS-deficient mice (supplemental Fig. S3A–G).

### Hepatic loss of GS accelerates hepatic steatosis progression and inflammation in MASLD mice

**Fig. 3:**
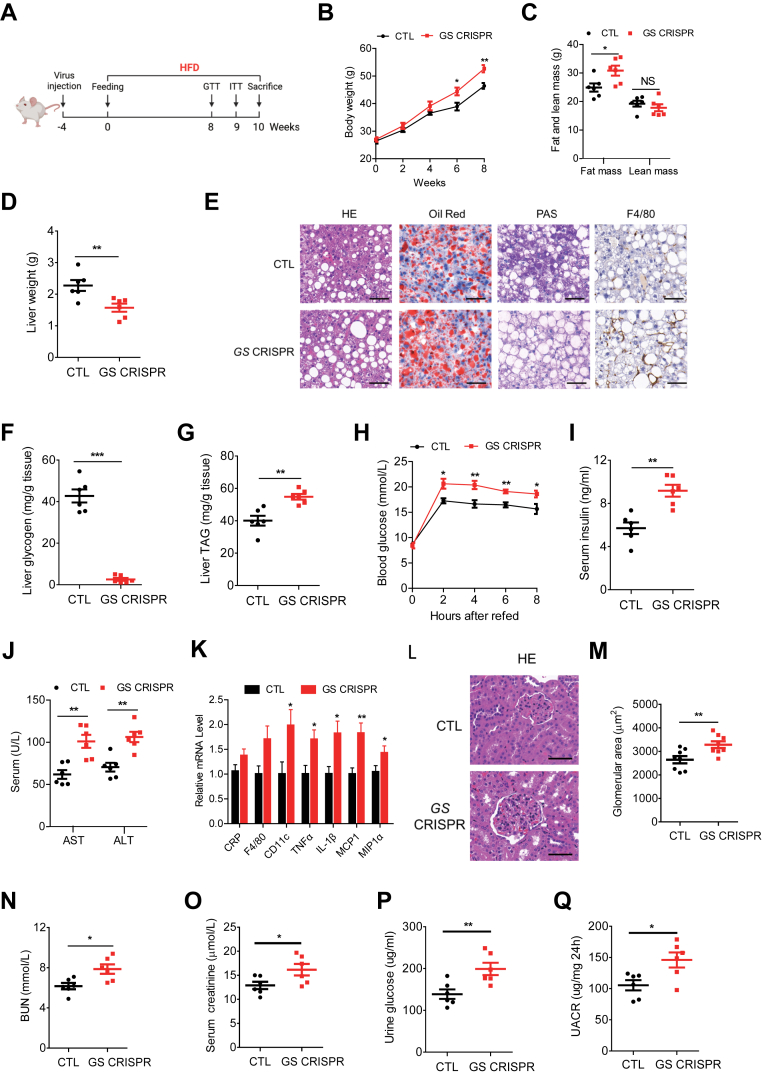
Loss of GS accelerates hepatic steatosis progression and inflammation in MASLD mice. A: Schematic diagram of a mouse model fed an HFD. B-D: Body weight, fat mass, lean mass, and liver weight of CTL and GS-deficient mice, respectively (n = 6). E: Representative images of liver sections stained with H&E, Oil Red O, PAS, and F4/80. The scale bars represent 50 μm. F and G: Determination of glycogen and TAG in liver tissues, respectively (n = 6). H: Blood glucose was measured in overnight-fasted CTL and GS-deficient mice at the indicated time points after refeeding (n = 6). I: Serum insulin was measured in overnight-fasted CTL and GS-deficient mice 8 h postprandial (n = 6). J: Serum AST and ALT (n = 6) measurements. K: Quantitative RT-PCR analysis of liver mRNA expression (n = 6). L: Representative images of kidney sections stained with H&E. The scale bars represent 50 μm. M: Quantitation of glomerular area of panel L. N and O: BUN and serum creatinine levels of CTL and GS-deficient mice (n = 6). P: Urinary glucose levels of CTL and GS-deficient mice (n = 6). Q: Urinary UACR levels of CTL and GS-deficient mice (n = 6). Data are presented as mean ± SEM. Data were analyzed using a two-tailed Student’s *t*-test. ∗*P* < 0.05, ∗∗*P* < 0.01, and ∗∗∗*P* < 0.001.

To explore whether an unhealthy diet could exacerbate pathologic changes in GS-deficient mice, we subjected control and GS-deficient mice to HFD for 8 weeks (Fig. 3A). A significant increase in body weight was observed in GS-deficient mice from week 6 onward, compared with control mice, despite no differences in food and water intake (Fig. 3B and supplemental Fig. S4A, B). The body weight gain in GS-deficient mice was probably because of an increase in fat mass (Fig. 3C). The liver weight of GS-deficient mice remained significantly lower than that of control mice (Fig. 3D). The complete absence of glycogen deposits was confirmed by PAS and biochemical analysis (Fig. 3E, F). H&E and Oil Red O staining revealed lipid accumulation, and biochemical measurements confirmed TAG accumulation in the liver of hepatic GS-deficient mice (Fig. 3E, G). Consistent with the phenotype observed in ND, blood glucose and serum insulin remained higher (Fig. 3 H, I) and GTT or ITT was significantly impaired in GS-deficient mice fed with HFD compared with the control group (supplemental Fig. S4C, D). Histochemistry, serum AST, ALT measurements, and inflammatory gene expression analysis increased in the liver tissues of GS-deficient mice, suggesting serious liver injury and lipid metabolism disorder (Fig. 3J, K). Further serum biochemistry analysis showed that inflammatory cytokines, TNFα and IL-6, were also significantly increased (supplemental Fig. S4E, F).

To determine the effect of hepatic GS deficiency on the structure of the glomerulus, H&E staining was conducted on kidney sections (Fig. 3L). Clearly, abnormal morphology in the glomeruli with an increase in the glomerular areas was determined in GS-deficient mice (Fig. 3M). Concomitantly, elevation in BUN, serum creatinine, urine glucose, and urine albumin-to-creatinine ratio (UACR) further indicate kidney dysfunction in GS-deficient mice (Fig. 3N–Q).

To directly test the requirement of hepatic GS in metabolic homeostasis, we performed rescue experiments by overexpressing GS in GS-deficient mice (supplemental Fig. S5A–C). Notably, reintroduction of GS largely reversed the reduced liver weight, the elevated liver TAG, and UACR mediated by hepatic GS inactivation (supplemental Fig. S5D–G). Together, we have further demonstrated the specificity of GS gene-editing effects reported in Fig. 3.

### The loss of GS promotes hepatic lipid accumulation via the fatty acid de novo synthesis pathway

**Fig. 4:**
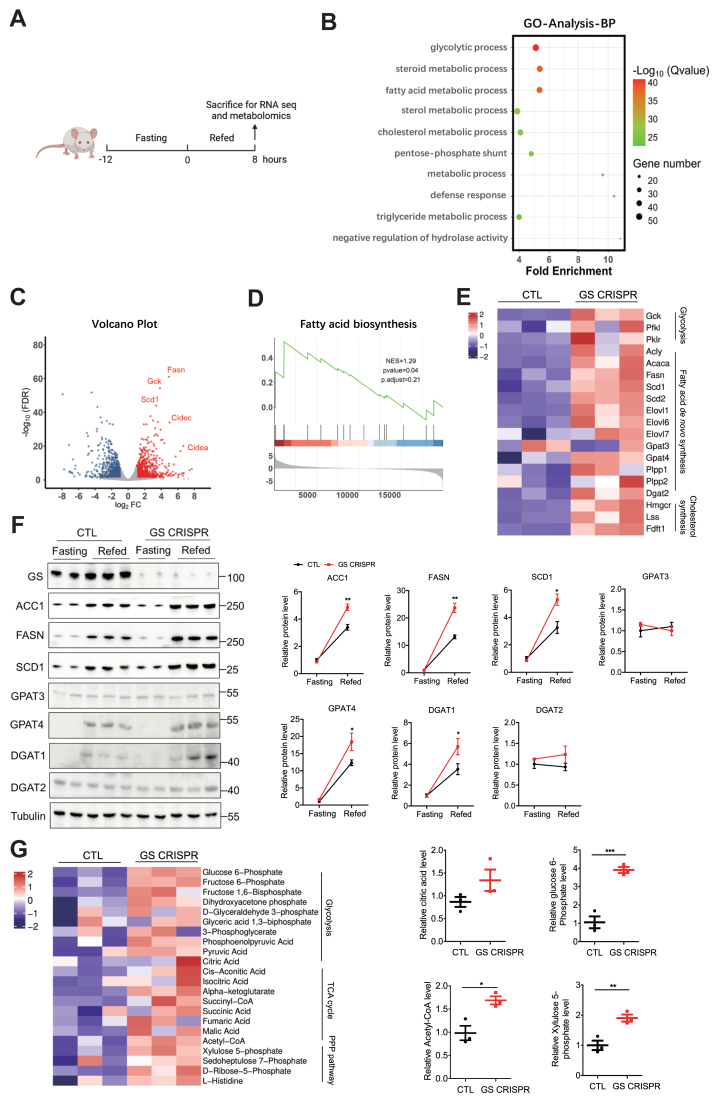
Loss of GS promotes lipid accumulation because of increased fatty acid de novo synthesis. A: Schematic diagram of the study design. RNA-Seq and metabolomics were performed on the livers of CTL and GS-deficient mice under ND condition (n = 3). B: Gene Ontology analysis of all significantly changed genes in the top 10 biological processes. C: Volcano plot representation of significantly upregulated and downregulated genes. D: Gene set enrichment analysis plot illustrating enrichment of the “Fatty acid biosynthesis signaling pathway” signature. E: Heatmap of significantly upregulated genes from RNA-Seq. F: Representative Western blots (left) and quantitative analysis (right panel) of liver samples obtained from CTL and GS-deficient mice with the indicated antibodies. G: Heatmap of liver metabolomics from CTL and GS-deficient mice (left), and the relative levels of citric acid, glucose 6-phosphate, acetyl-CoA, and xylulose 5-phosphate (right). Data are presented as mean ± SEM. Data were analyzed using a two-tailed Student’s *t*-test. ∗*P* < 0.05, ∗∗*P* < 0.01, and ∗∗∗*P* < 0.001.

To comprehensively investigate the role of GS deficiency in fat accumulation, we performed RNA-Seq and metabolomics analysis of liver samples from ND-fed control and GS-deficient mice after refeeding (Fig. 4A). Gene Ontology analysis indicated that glycolytic process and fatty acid metabolic process were remarkably upregulated in GS-deficient mice (Fig. 4B). The volcano plot showed that genes involved in glycolysis including glucokinase and fatty acid de novo synthesis, such as *FASN* and *SCD1*, and LD synthesis, such as cell death inducing DFFA-like effector C (*Cidec*) and cell death-inducing DFFA-like effector A (*Cidea*), were upregulated in GS-deficient mice (Fig. 4C). Gene set enrichment analysis revealed that the fatty acid de novo synthesis pathway was significantly upregulated in GS-deficient mice (Fig. 4D and E). Several genes selected from the dataset were independently validated by RT-PCR and immunoblotting and found to be significantly activated after refeeding in GS-deficient liver tissues. These include FASN, ACC1, SCD1, GPAT4, and DGAT1 (Fig. 4F and supplemental Fig. S6A–C).

Notably, metabolomics analysis showed that compared with control mice, glycolysis, tricarboxylic acid cycle, and pentose phosphate pathway were significantly increased in GS-deficient mice (Fig. 4G). Here, citric acid was found to increase slightly in GS-deficient mice (Fig. 4G). More importantly, several glucose-derived metabolites, including glucose 6-phosphate, acetyl-CoA, and xylulose 5-phosphate, which are potential activators of ChREBP, were substantially elevated in GS-deficient mice (Fig. 4G) (25). Collectively, these data suggest that GS ablation in the liver promotes substrate accumulation derived from glycolysis for lipid synthesis.

### Hepatic GS deficiency leads to elevated fatty acid synthesis via the ChREBP and AKT-mTOR1-SREBP1 pathway

**Fig. 5:**
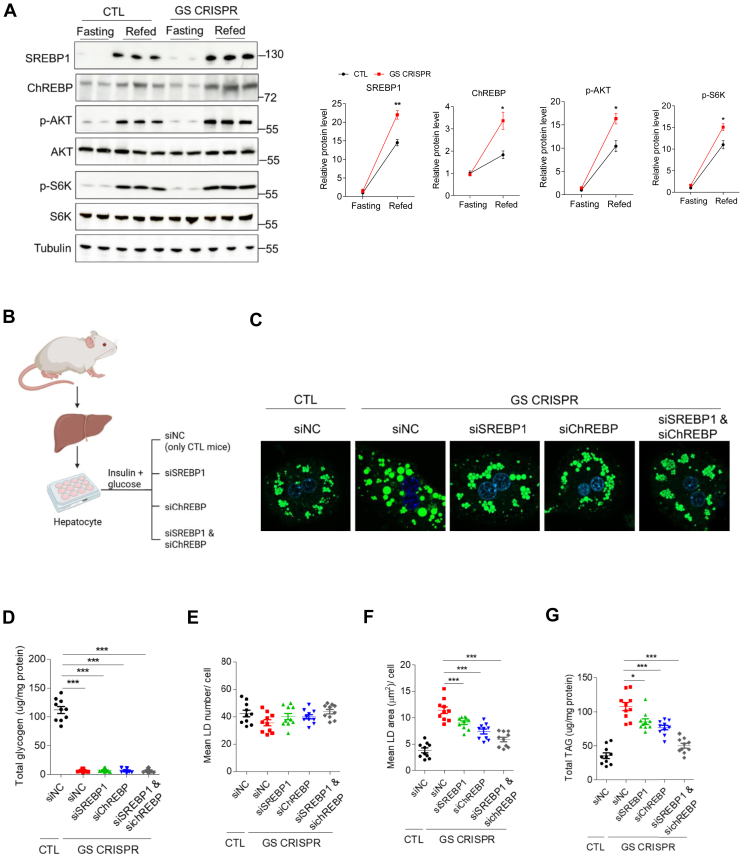
GS deficiency activates ChREBP and AKT-mTORC1-SREBP1 pathways. A: Representative Western blots (left) and quantitative analysis (right panel) of liver samples obtained from CTL and GS-deficient mice with the indicated antibodies. B: Schematic diagram of the study design. Hepatocytes isolated from CTL mice were transfected with nontargeting siRNA controls (siNC); hepatocytes isolated from GS-deficient mice were transfected with one of the following four siRNAs: siNC, siRNA targeting SREBP1 (siSREBP1), ChREBP (siChREBP), or both (siSREBP1 + siChREBP). C: Representative images of LDs (*green*) in hepatocytes isolated from CTL and GS-deficient mice. LDs were labeled with BODIPY (*green*), and the nucleus was labeled with DAPI (*blue*). The scale bars represent 10 μm. The diameter of LDs was measured from at least 20 cells. D–G: Histograms showing total glycogen, mean LD number, mean LD area, and total TAG in C. Data are presented as mean ± SEM. Data were analyzed using a two-tailed Student’s *t*-test. ∗*P* < 0.05, ∗∗*P* < 0.01, and ∗∗∗*P* < 0.001.

It has been well established that in response to hormonal and nutritional stimuli, two transcription factors are involved in fatty acid de novo synthesis: SREBP1 in response to insulin and ChREBP in response to carbohydrates (25). Interestingly, loss of hepatic GS led to a significant increase in ChREBP and AKT-mTOR1-SREBP1 pathway (Fig. 5A). However, in response to insulin injection, Akt phosphorylation, as well as its downstream target, p-GSK3β, was modestly but significantly lowered in the liver (supplemental Fig. S7A) of GS-deficient mice. No differences were observed in the muscle and WAT of control and GS-deficient mice (supplemental Fig. S7B, C). These data suggest an impaired hepatic insulin signaling in GS-deficient mice.

To further explore the role of SREBP1 and ChREBP pathways in fat accumulation, we transfected hepatocytes isolated from the livers of control and GS-deficient mice with nontargeting siRNA controls (siNC), siRNA targeting SREBP1 (siSREBP1), ChREBP (siChREBP), or both (siSREBP1 + siChREBP) (Fig. 5B). Western blot analysis confirmed that siRNA knockdown efficiently revert SREBP1 and ChREBP protein levels in hepatocytes from GS-deficient mice to the levels observed in CTL hepatocytes (supplemental Fig. S8A). To verify that siRNA knockdown reduced the activity (and not just the expression) of SREBP1 and ChREBP, we measured the mRNA levels of their canonical target genes (*ACC1*, *FASN*, and *SCD1*) by RT-PCR. As expected, siRNA knockdown significantly suppressed the expression of these target genes (*P* < 0.05), confirming the functional inhibition of both transcription factors (suplemental Fig. S8B). Next, the various siRNA-targeted hepatocytes were treated with high glucose and insulin to induce lipogenesis, followed by Bodipy staining to quantify LDs, and cellular TAG was measured (Fig. 5C). As expected, glycogen remained almost undetectable in primary hepatocytes isolated from GS-deficient mice regardless of SREBP1 and ChREBP knockdown (Fig. 5D). Mean LD number did not differ significantly among the above treatments (Fig. 5E). However, treatment of siSREBP1 or siChREBP on GS-deficient hepatocytes reversed both mean LD area and total TAG levels especially when double knockdown was performed (Fig. 5F, G). Notably, mean LD area and total TAG were significantly lower in siChREBP-treated hepatocytes compared with those of siSREBP1 (Fig. 5F, G). Collectively, our findings demonstrate that ChREBP serves as the principal regulator of fat accumulation, with SREBP1 exerting secondary effects.

### AAV-mediated restoration of hepatic GS decreases fat accumulation in *db/db* mice

**Fig. 6:**
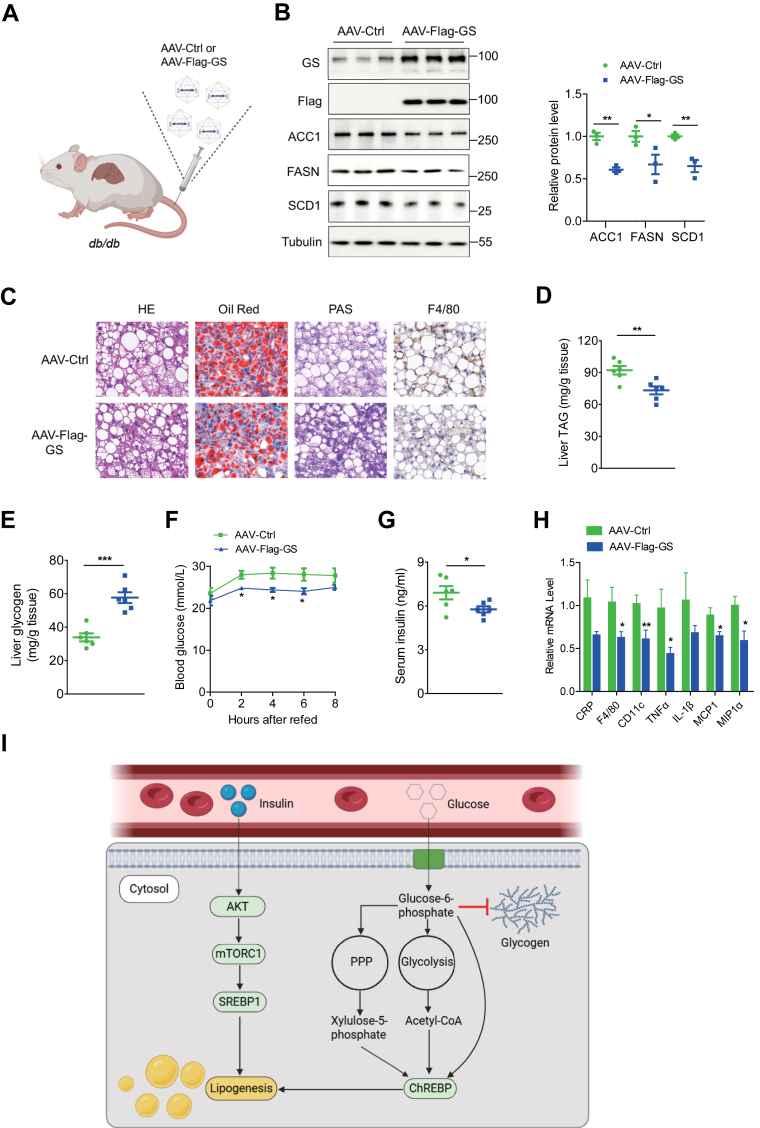
Overexpression of hepatic GS in *db/db* mice reduces lipid accumulation. A: Schematic diagram showing the AAV8-TBG-Ctrl or AAV8-TBG-FLAG-GS administration protocol in *db/db* mice (n = 6/group). B: Representative Western blots (left) and quantitative analysis (right panel) of hepatic GS overexpression. C: Representative images of liver sections stained with H&E, Oil Red O, PAS, and F4/80. The scale bars represent 50 μm. D and E: Determination of TAG and glycogen in liver tissues (n = 6). F: Blood glucose mice were measured in overnight-fasted animals at the indicated time points after refeeding (n = 6). G: Serum insulin was measured in overnight-fasted animals 8 h postprandial (n = 6). H: Quantitative RT-PCR analysis of liver mRNA expression (n = 6). I: Schematic diagram illustrating the role of GS deficiency in lipogenesis. In GS-deficient mice, postprandial impairment of glycogen synthesis elicits a dual metabolic response: on one hand, hyperglycemia and subsequent hyperinsulinemia activate the mTOR-SREBP1 axis; on the other hand, elevated intrahepatic glucose diverts metabolic flux toward glycolysis and the pentose phosphate pathway, increasing xylulose-5-phosphate and acetyl-CoA, which subsequently activate the ChREBP pathway. These two mechanisms ultimately converge to drive enhanced hepatic lipogenesis. Data are presented as mean ± SEM. Data were analyzed using a two-tailed Student’s *t*-test. ∗*P* < 0.05 and ∗∗*P* < 0.01.

Next, we investigated whether GS overexpression could rescue the fat-accumulation phenotype in *db/db* mice. In this study, *db/db* mice were injected with an AAV8 vector expressing GS under the control of a liver-specific promoter (TBG). AAV8-TBG-ctrl or AAV8-TBG-FLAG-GS was introduced through tail vein injection (Fig. 6A). The overexpression of the GS protein was confirmed with immunoblotting. Interestingly, high GS protein expression in *db/db* mice led to a significant reduction in ACC1, FASN, and SCD1 protein levels (Fig. 6B). In addition, TAG levels were significantly reduced (Fig. 6C, D). Further analysis showed that increasing hepatic GS expression elevated hepatic glycogen levels (Fig. 6C, E). Moreover, mice overexpressing GS exhibited lower blood glucose and insulin levels after refeeding compared with controls (Fig. 6F and G). Furthermore, overexpression of hepatic GS resulted in a modest yet significant reduction in the mRNA expression of genes associated with inflammation (Fig. 6H). Taken together, these data support that augmented GS expression in the liver confers protection against fat accumulation in mice (Fig. 6I).

## Discussion

Our study demonstrates that the hepatic glycogenesis enzyme GS plays an important role in the development and pathogenesis of MASLD. The GS protein expression is reduced in the livers of HFD-fed mice or *db/db* mice. Mechanistically, loss of GS promotes steatosis by enhancing lipid deposition through the activation of ChREBP and SREBP1, thus promoting MASLD progression. Importantly, hepatic overexpression of GS in *db/db* mice significantly alleviates the metabolic dysfunction, offering a potential therapeutic strategy to treat MASLD.

The apparent paradox of reduced liver weight despite an increase in hepatic steatosis is likely to stem from the profound loss of glycogen and its associated water weight (26). Glycogen is highly hygroscopic, and its near-complete depletion in the liver of GS CRISPR mice causes a substantial loss of mass that outweighs the gain from lipid accumulation (13). Furthermore, the lower density of lipid storage compared with the glycogen-water complex may further decouple steatosis from weight gain. The metabolic stress arising from disrupted energy homeostasis may also impact hepatocyte size or proliferation capacity, and this possibility requires further in-depth investigation.

Glycogenesis and lipogenesis are two fundamental pathways that cells use to store energy (13, 27, 28). Upon feeding, elevated blood glucose induces insulin secretion from the pancreas, which enhances glucose uptake into the peripheral tissues, such as the liver and skeletal muscles. Excess glucose molecules are transported into the hepatocytes to be stored either as glycogen via glycogenesis or fat via glycolysis-TCA-lipogenesis flux (29, 30, 31). The liver is the central regulator of postprandial blood glucose homeostasis, and the tissue serves to dispose of as much as one-third of the oral glucose load (32, 33). Dysregulation of postprandial hepatic glucose metabolism promotes the development of metabolic disorders, including insulin resistance and type 2 diabetes (34).

In this present study, inhibition of hepatic glycogenesis leads to systemic metabolic dysregulation across multiple pathways. First, GS-deficient mice exhibited persistently elevated blood glucose levels after food consumption, leading to markedly increased serum insulin levels. When the mice were injected with an equivalent amount of insulin, we found that the in vivo insulin-induced response diminished in the liver of GS-deficient mice. This is consistent with data previously reported by Irimia *et al.* (16). The impaired insulin sensitivity is largely attributed to exacerbated lipid accumulation and inflammation in the liver of GS-deficient mice. However, it should be noted that significantly elevated serum insulin levels after food consumption not only counteract the deleterious effects of hepatic insulin resistance but also further potentiate PI3K-AKT-mTORC1 signaling pathway activation. This ultimately activates the SREBP1 pathway, thereby inducing de novo lipogenesis via coordinated upregulation of lipogenic genes.

Second, the inhibition of hepatic glycogenesis resulted in an accumulation of glucose in the livers of GS-deficient mice, which promotes glucose flux into the glycolytic and pentose phosphate pathways. Subsequently, metabolites, such as glucose 6-phosphate, acetyl-CoA, and xylulose 5-phosphate, in turn activate ChREBP, which promotes lipid biosynthesis through transcriptional induction of lipogenic genes (25). Furthermore, these elevated metabolites provide abundant substrates for lipid biosynthesis.

Third, our findings strongly support the conclusion that upregulation of SREBP1 and ChREBP is the primary driver of elevated lipid accumulation observed in our model. This is directly evidenced by the observation that siRNA-mediated suppression of SREBP1 or ChREBP effectively abolished TAG increase, underscoring their essential role in mediating lipogenic flux. However, it is important to acknowledge the significant role of substrate availability. Even when elevation in SREBP1 and ChREBP was suppressed, a residual, albeit lower, level of TAG accumulation persisted compared with the control. This observation supports the suggestion whereby failure to store glucose as glycogen creates a metabolic "overflow," channeling more glucose carbon through glycolysis and into the lipogenic pathway. Therefore, while SREBP1 and ChREBP are critical amplifiers of hepatic steatosis, the underlying metabolic perturbation—diversion of glucose from glycogen synthesis—provides a fundamental drive for the increase in lipid accumulation.

Our comprehensive data robustly demonstrate that hepatic GS deficiency exacerbates kidney injury under HFD conditions. The observed renal pathology, characterized by significant glomerular hypertrophy and elevated kidney functional markers (UACR, serum creatinine, and BUN), provides conclusive evidence of impaired renal function. The mechanistic link appears to be primarily driven by the systemic metabolic consequences of GS ablation. Specifically, GS KO led to pronounced hyperglycemia, which acts as a primary driver of glomerular stress and injury, a hallmark of diabetic nephropathy (35, 36). Furthermore, this metabolic perturbation is compounded by a state of systemic inflammation, as indicated by significant elevation in serum proinflammatory cytokines, TNFα and IL-6. The combination of chronic hyperglycemia and proinflammatory milieu potentially synergizes to drive glomerular damage and dysfunction, thereby accelerating kidney injury in this model.

In conclusion, hepatic GS deficiency promotes lipid deposition in normal- or HFD-fed male mice. Importantly, hepatic GS loss activates ChREBP and AKT-mTORC1-SREBP1 pathway, which in turn increases fatty acid de novo synthesis in the male mice. Overexpression of hepatic GS reduces lipid accumulation, thus providing a potential therapeutic strategy for MASLD treatment.

## Data availability

Data are available from the corresponding author upon reasonable request.

## Supplemental data

This article contains supplemental data.

## Conflict of interest

The authors declare that they have no conflicts of interest with the contents of this article.

## Supplemental Data

Sup figure 1

Sup figure 2

Sup figure 3

Sup figure 4

Sup figure 5

Sup figure 6

Sup figure 7

Sup figure 8
