# Supplementary figures and images for: Deficiency of glycogen synthase promotes lipid accumulation through ChREBP and AKT-mTOR1-SREBP1 axis activation in mice
Source: J Lipid Res. 2025 Dec 15;67(1):100962. doi: 10.1016/j.jlr.2025.100962 (PMC12818132; doi:10.1016/j.jlr.2025.100962)

A

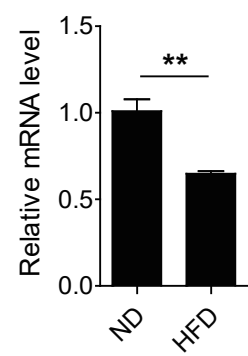

B

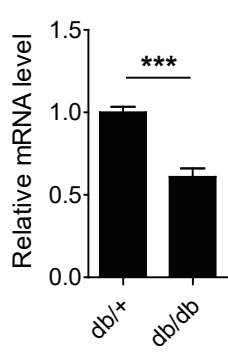

Supplement: Sup figure 1 [file mmc1.pdf]

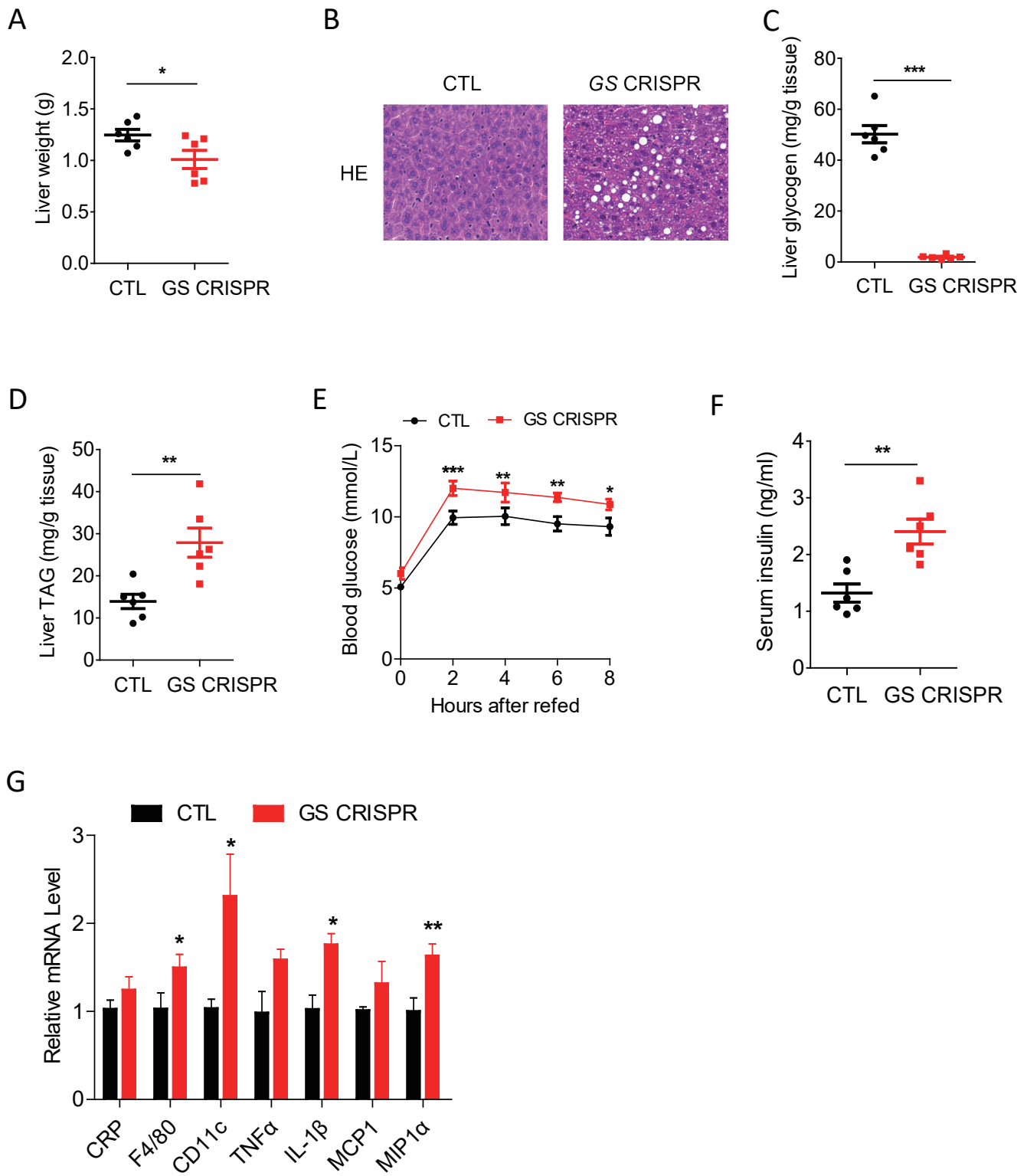

Supplement: Sup figure 3 [file mmc3.pdf]

A

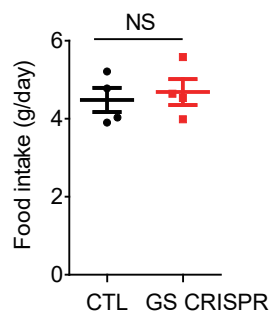

B

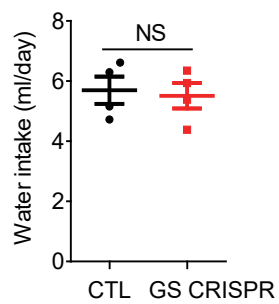

C

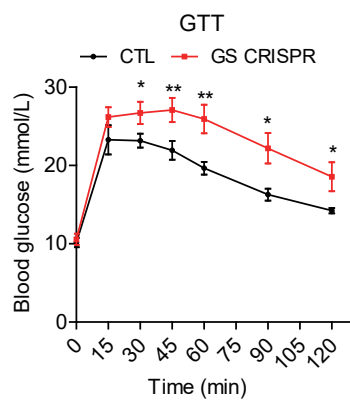

D

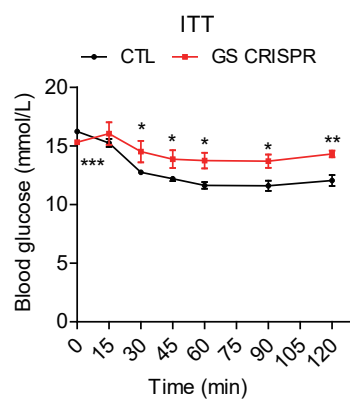

E

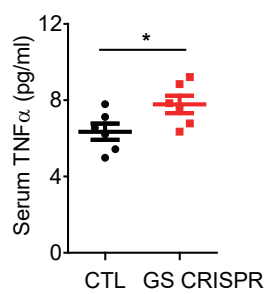

F

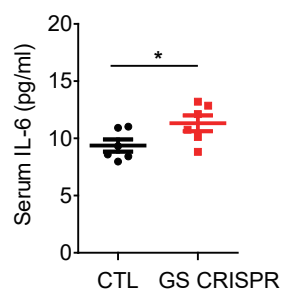

Supplement: Sup figure 4 [file mmc4.pdf]

A

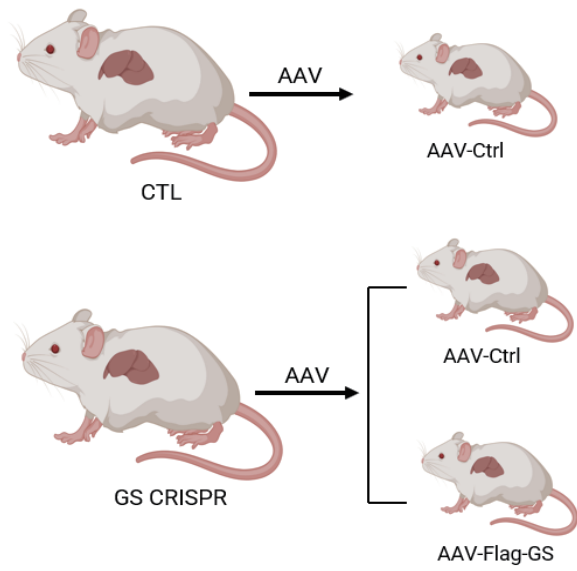

B

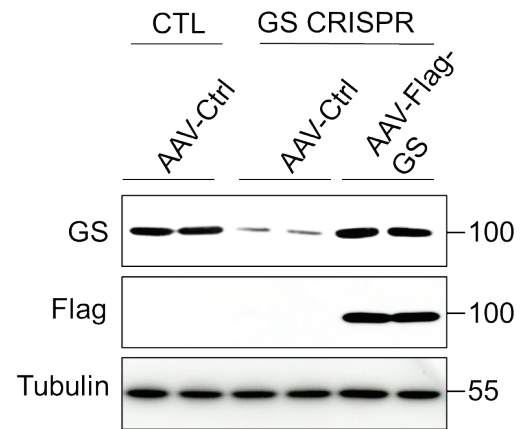

C

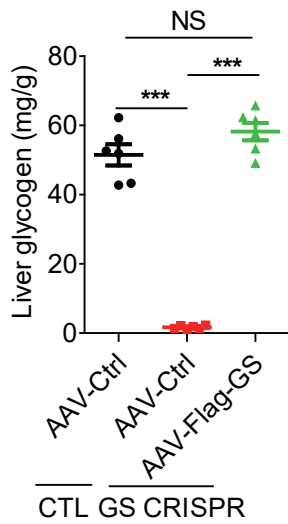

D

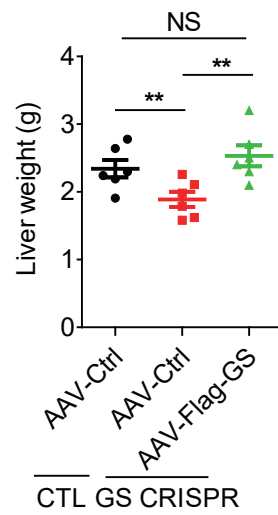

E

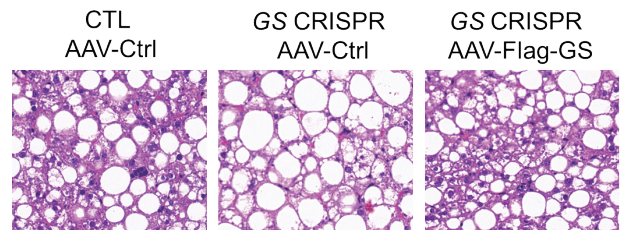

F

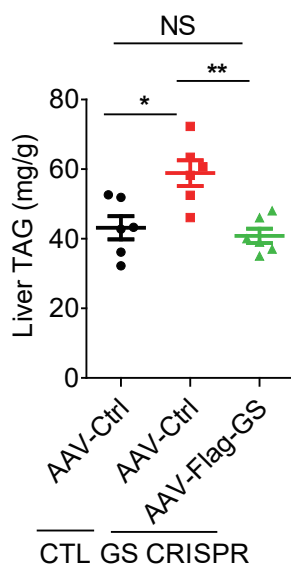

G

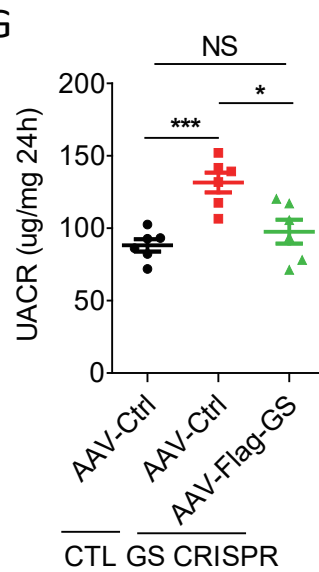

Supplement: Sup figure 5 [file mmc5.pdf]

**A**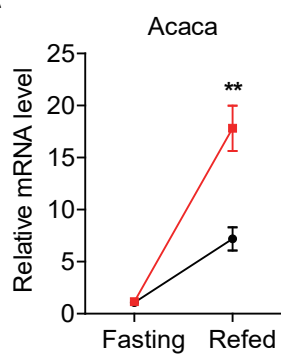**B**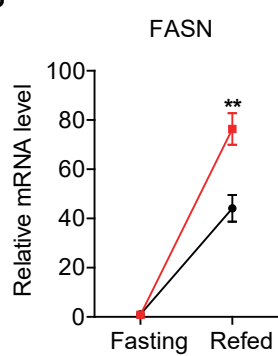**C**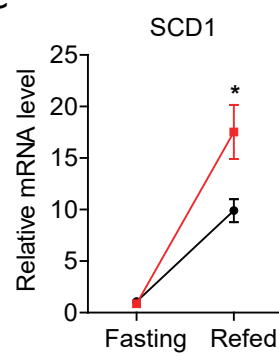

Supplement: Sup figure 6 [file mmc6.pdf]

**A**

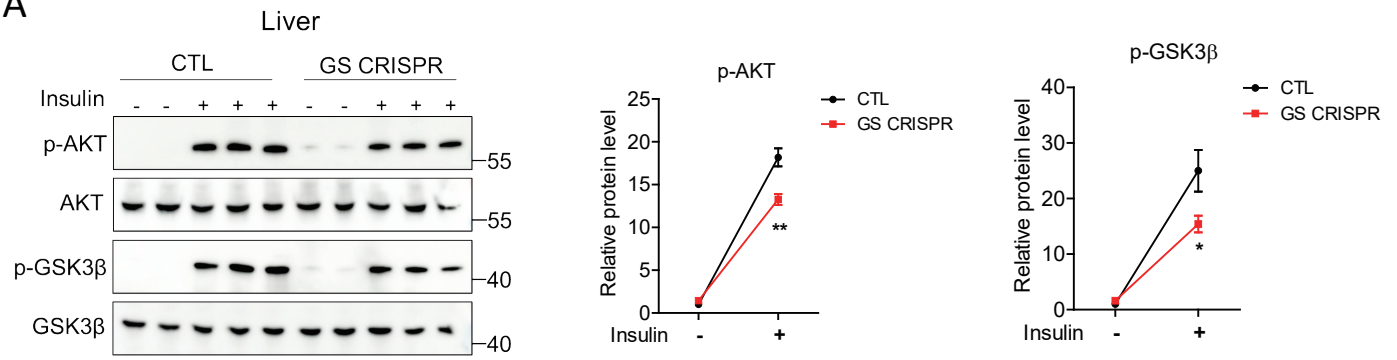

**B**

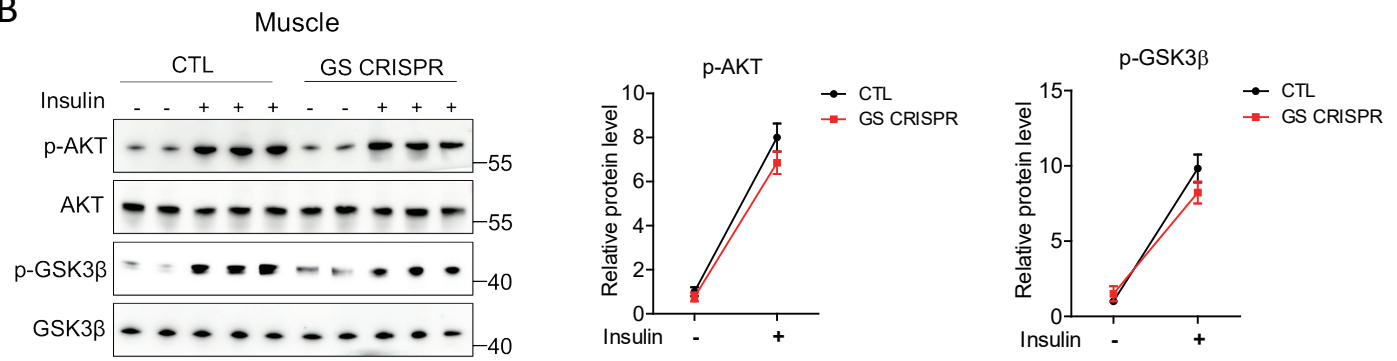

**C**

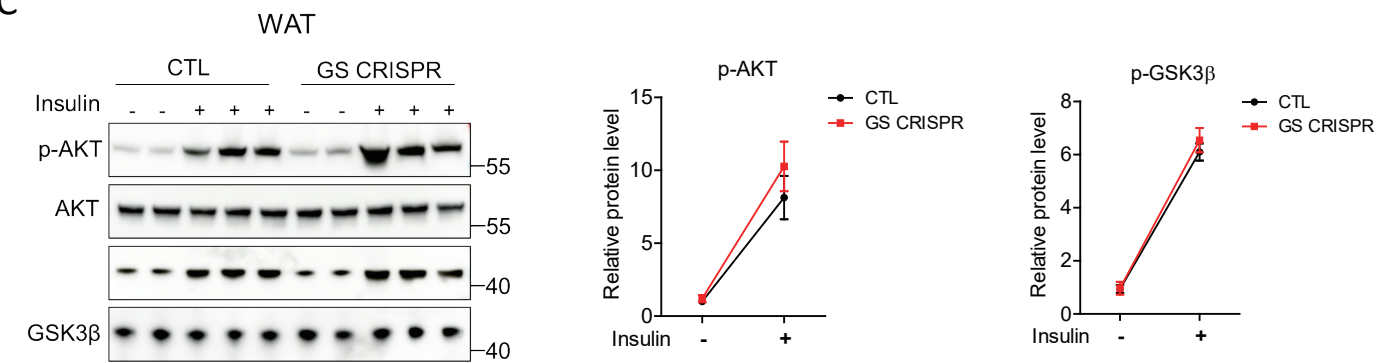

Supplement: Sup figure 7 [file mmc7.pdf]

A

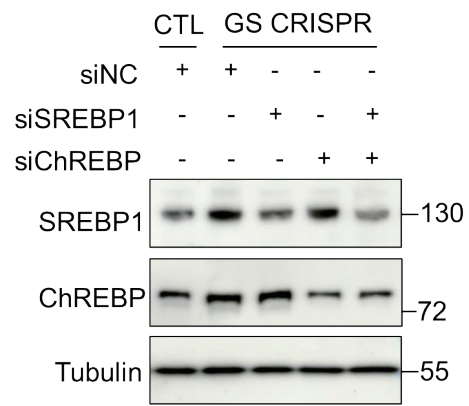

B

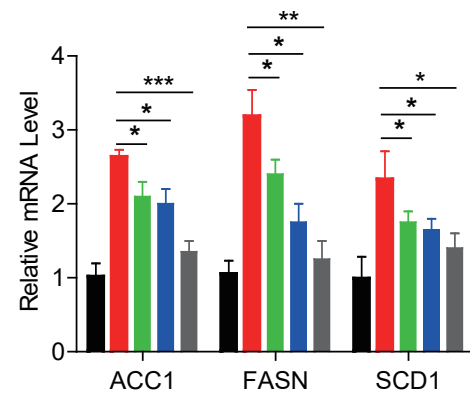

Supplement: Sup figure 8 [file mmc8.pdf]
