# Supplementary material for: Deficiency of glycogen synthase promotes lipid accumulation through ChREBP and AKT-mTOR1-SREBP1 axis activation in mice
Source: J Lipid Res. 2025 Dec 15;67(1):100962. doi: 10.1016/j.jlr.2025.100962 (PMC12818132; doi:10.1016/j.jlr.2025.100962)

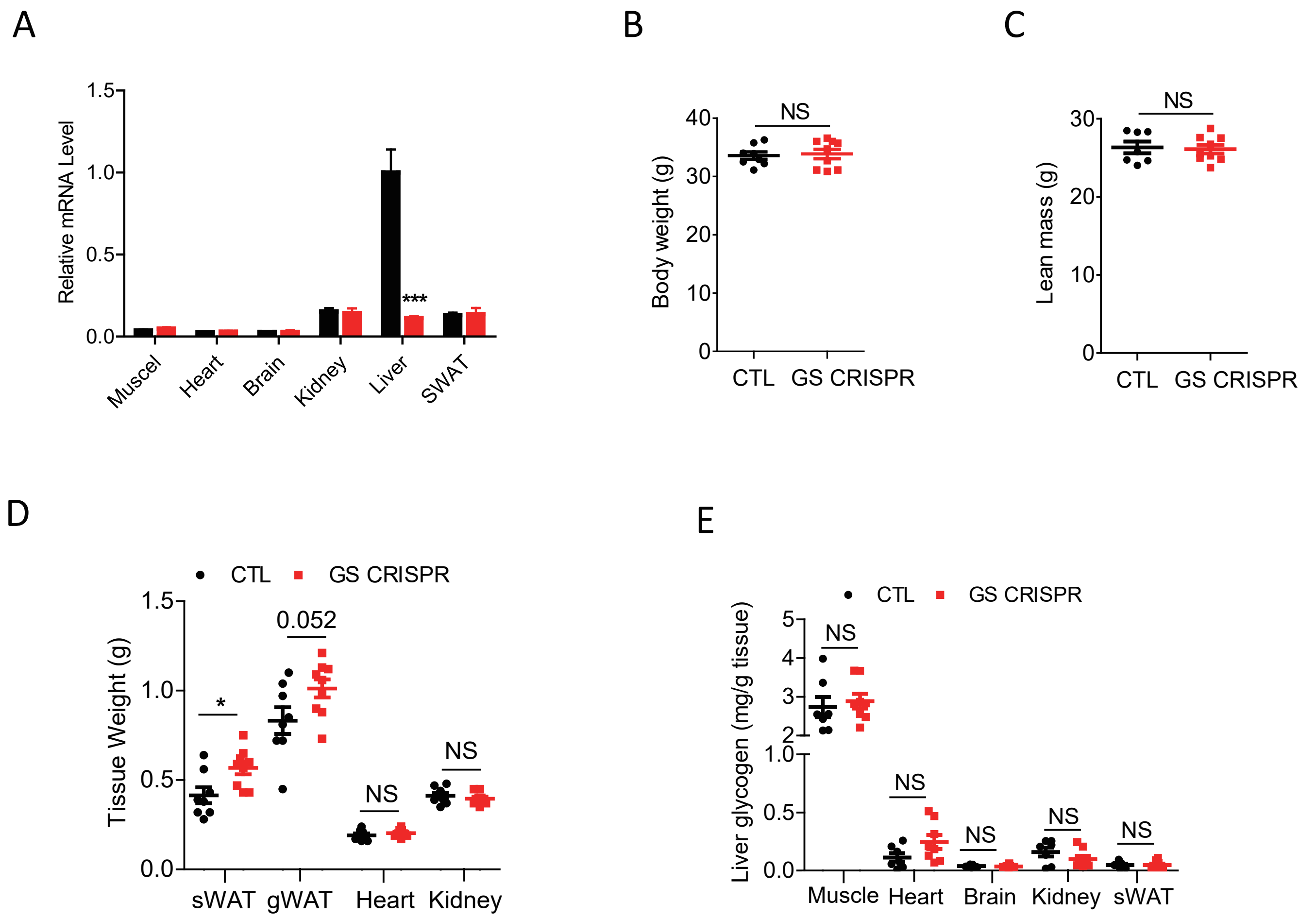

F

Parameters	CTL<br>Mean ± SEM<br>n=8	*GS* CRISPR<br>Mean ±SEM<br>n=8	p-value
Fed NEFA (mEq/L)	0.38 ± 0.02	0.46 ± 0.03	0.02*
Fasted NEFA (mEq/L)	0.76 ± 0.08	1.14 ± 0.11	0.017*
Fed TAG (mg/ml)	0.73 ± 0.07	0.92 ± 0.06	0.07
Fasted TAG (mg/ml)	0.64 ± 0.07	0.86 ± 0.07	0.046*
Fed cholesterol (mg/dL)	119.62 ± 4.10	117.11 ± 4.21	0.68
Fasted cholesterol (mg/dL)	107.96± 2.98	113.01 ± 2.78	0.25

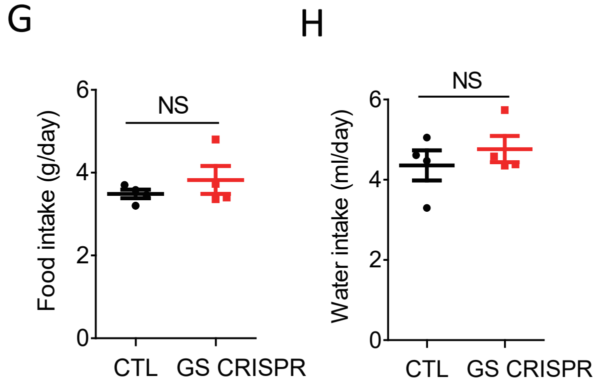

Supplement: Sup figure 2 [file mmc2.pdf]
