# Supplementary material for: Deficiency of glycogen synthase promotes lipid accumulation through ChREBP and AKT-mTOR1-SREBP1 axis activation in mice
Source: J Lipid Res. 2025 Dec 15;67(1):100962. doi: 10.1016/j.jlr.2025.100962 (PMC12818132; doi:10.1016/j.jlr.2025.100962)
Supplement: Supplementary table 1 [file mmc9.docx]

Supplementary Table 1. Sequences of oligonucleotides for sgRNA constructs

|  | Guide sequence (5’-3’) |
| --- | --- |
| LacZ-sg | TGCGAATACGCCCACGCGAT |
| Gys2-sg | GATGTCAAAGAGCACCACGT |
