# Supplementary material for: Deficiency of glycogen synthase promotes lipid accumulation through ChREBP and AKT-mTOR1-SREBP1 axis activation in mice
Source: J Lipid Res. 2025 Dec 15;67(1):100962. doi: 10.1016/j.jlr.2025.100962 (PMC12818132; doi:10.1016/j.jlr.2025.100962)
Supplement: Supplementary table 2 [file mmc10.docx]

Supplementary Table 2. Sequences of primers used for RT-PCR analysis.

|  | Forward (5’-3’) | Reverse (5’-3’) |
| --- | --- | --- |
| Actin | GGCTGTATTCCCCTCCATCG | CCAGTTGGTAACAATGCCATGT |
| Gys2 | ACCAAGGCCAAAACGACAG | GGGCTCACATTGTTCTACTTGA |
| CRP | ATGGAGAAGCTACTCTGGTGC | ACACACAGTAAAGGTGTTCAGTG |
| F4/80 | CTTTGGCTATGGGCTTCCAGTC | GCAAGGAGGACAGAGTTTATCGTG |
| CD11c | ACACAGTGTGCTCCAGTATGA | GCCCAGGGATATGTTCACAGC |
| TNFα | CCAGACCCTCACACTCAGATC | CACTTGGTGGTTTGCTACGAC |
| IL-1β | GCAACTGTTCCTGAACTCAACT | ATCTTTTGGGGTCCGTCAACT |
| MCP1 | AGGTCCCTGTCATGCTTCTG | GCTGCTGGTGATCCTCTTGT |
| MIP1α | TTCTCTGTACCATGACACTCTGC | CGTGGAATCTTCCGGCTGTAG |
| Acaca | AGCTGATCCTGCGAACCT | GCCAAGCGGATGTAAACT |
| FASN | TCCAAGACTGACTCGGCTACTGAC | GCAGCCAGGTTCGGAATGCTATC |
| SCD1 | TTCTTGCGATACACTCTGGTGC | CGGGATTGAATGTTCTTGTCGT |
