# Supplementary material for: Deficiency of glycogen synthase promotes lipid accumulation through ChREBP and AKT-mTOR1-SREBP1 axis activation in mice
Source: J Lipid Res. 2025 Dec 15;67(1):100962. doi: 10.1016/j.jlr.2025.100962 (PMC12818132; doi:10.1016/j.jlr.2025.100962)
Supplement: Supplementary table 3 [file mmc11.docx]

Supplementary Table 3. siRNAs used in this work.

| Target gene | siRNA sequences (5′–3′) |
| --- | --- |
| SREBP1 | GCAUUGAGACCUGGUUCAA |
| ChREBP | GGACCUACACCAAGAGCAATT |
